# Radiocaesium Contamination of Mushrooms at High- and Low-Level Chernobyl Exposure Sites and Its Consequences for Public Health

**DOI:** 10.3390/life11121370

**Published:** 2021-12-09

**Authors:** Ondřej Harkut, Petr Alexa, Radim Uhlář

**Affiliations:** Department of Physics, Faculty of Electrical Engineering and Computer Science, VŠB-Technical University of Ostrava, 17. listopadu 2172/15, 708 00 Ostrava, Czech Republic; ondrej.harkut.st@vsb.cz (O.H.); radim.uhlar@vsb.cz (R.U.)

**Keywords:** radiocaesium, mushrooms, Chernobyl accident

## Abstract

We compare the specific activities of ${}^{137}$Cs and ${}^{40}$K in stipes and caps of three different common mushroom species (*Xerocomus badius*, *Russula ochroleuca* and *Armillariella mellea*) measured at the Czech Chernobyl hot spot in the Opava area (Silesia) and at a low-exposed site at the Beskydy mountains in 2011. The highest values of ${}^{137}$Cs were found in caps of *Xerocomus badius* and *Russula ochroleuca* in the Opava area (11.8 and 8.77 kBq/kg, respectively). The source of ${}^{137}$Cs was verified by the measurement of the ${}^{134}$Cs/${}^{137}$Cs ratio. Based on our results, we estimate an effective dose per year due to radiocaesium intake in the two investigated areas for *Xerocomus badius*, one of the most popular edible mushrooms in the Czech Republic. In 2011, the effective dose reached the maximum value of 0.102 mSv in the Opava area and 0.004 mSv at the low-exposed site at the Beskydy mountains. Therefore, it does not represent a significant risk for public health.

## 1. Introduction

Wild fungi and their fruiting bodies—so-called mushrooms—tend to accumulate radiocaesium that represents a problematic environmental issue particularly due to a relatively long half-life, emission of gamma radiation and high risk of incorporation into living organisms [1,2,3,4]. This effect has already been examined closely over decades after pollution events [2,5]. Due to the impact of nuclear weapon tests, the Chernobyl accident in 1986 [4,6,7] and the Fukushima accident in 2011 [8], soils around the globe are contaminated by radiocaesium [9].

The soil-to-fungi transfer causes the accumulation of larger amounts of ${}^{137}$Cs in wild mushrooms depending on type of soil and its surface activity of radiocaesium [10,11,12,13,14]. Wild edible mushrooms in Czech forest ecosystems are commonly picked and eaten by dwellers, which represents a risk of receiving additional effective doses by ingesting higher levels of ${}^{137}$Cs than recommended by IAEA [15].

The IAEA recommendation suggests the generic action level for ${}^{137}$Cs of 1 kBq/kg. If the specific activity exceeds the level, an action of some sort should be taken. Simultaneously, the IAEA recommendation states that classes of food that are consumed in small quantities, e.g., less than 10 kg per person per year, which represent a very small fraction of the total diet and would make very small additions to individual exposures, may have action levels ten-times higher than those for major foodstuffs.

Measurements of ${}^{137}$Cs specific activity in different parts of mushrooms (caps and stipes—in some cases gills and pores) have been carried out at particular areas in the Slovak Republic [16], in Poland [1,17,18], in Austria [19] and in southern Germany [20]. The studies [21,22] dealt with this issue in the Czech Republic. All these countries have been affected by a radioactive cloud from Chernobyl. Mountain areas are susceptible to rainfalls that are able to release particulates from radioactive clouds into a forest environment [23].

The measured specific activities greatly depend on the amount of precipitations that were absorbed by soil. This effect created high- and low-level Chernobyl exposure sites across the country.

Mushrooms are characterized by a high ability to accumulate radiocaesium and work well as bioindicators of radioactivity in nature [24]. The reason lies within their structure, which consists of gentle fibres. The genetic constitution of mushrooms differs from green plants that absorb caesium less efficiently than its nutrient element, potassium. The so-called Cs/K discriminator factor (DF) at mushrooms indicates the transportation efficiency of these elements within the mushroom structure, e.g., from stipe to cap [16,17,25,26,27,28,29,30].

The aim of our paper is to compare specific activities of ${}^{137}$Cs in mushrooms from two areas in the eastern part of the Czech Republic with different total precipitation amounts from the radioactive Chernobyl cloud that passed the areas on 30 April/1 May 1986. In the Opava region (Silesia) the total precipitation amount exceeded 15 mm, while in the Ostravice river valley in the Beskydy mountains, it was lower than 0.5 mm [6]. This resulted in a different initial surface activity in both areas.

The Chernobyl hot spot in the Opava region has not yet been examined in terms of the content of ${}^{137}$Cs in mushrooms in spite of the fact that the fallout from the radioactive cloud from the Chernobyl accident was one of the largest in the Czech Republic. Activity levels of ${}^{137}$Cs reached up to 52 kBq/m${}^{2}$ in soil samples [6]. We also tested a possibility to determine both ${}^{137}$Cs and ${}^{40}$K activity in caps and stipes for small samples (masses around 1 g and less) using a low-background well HPGe spectrometer.

Different parts of the fruitbody (caps and stipes) of the collected specimens (*Xerocomus badius*, *Russula ochroleuca* and *Armillariella mellea*) were analysed. The species *Xerocomus badius* was chosen as a commonly used reference edible mushroom for its high ability to accumulate radiocaesium [1], and a potential radiation risk due to high consumption of this species was determined.

## 2. Materials and Methods

In October and November 2011, fruiting bodies of three commonly used reference edible mushrooms (*Xerocomus badius*, *Russula ochroleuca* and *Armillariella mellea*) were collected from a square area of approximately 2.6 km${}^{2}$ in the Opava area (GPS coordinates of the centre of the area: $49^{\circ }52^{\prime }26.432^{\prime \prime }$ N, $18^{\circ }0^{\prime }30.972^{\prime \prime }$ E) and from a similar square area in the Ostravice river valley in the Beskydy mountains (GPS coordinates of the centre of the area: $49^{\circ }30^{\prime }0.825^{\prime \prime }$ N, $18^{\circ }26^{\prime }48.283^{\prime \prime }$ E).

The Chernobyl hot spot area in the Opava region where the collecting of mushrooms took place is located on a geological bedrock consisting of paleozoic predominantly sedimentary rocks (shale, greywacke, quartzite and limestone) whereas the geological bedrock in the Ostravice river valley consists of mezozoic sedimentary rocks (sandstone and shale). In the Opava region, sandy-loam brown soils prevail, while, in the Ostravice river valley, acid loam brown soils dominate.

**Figure 1:**
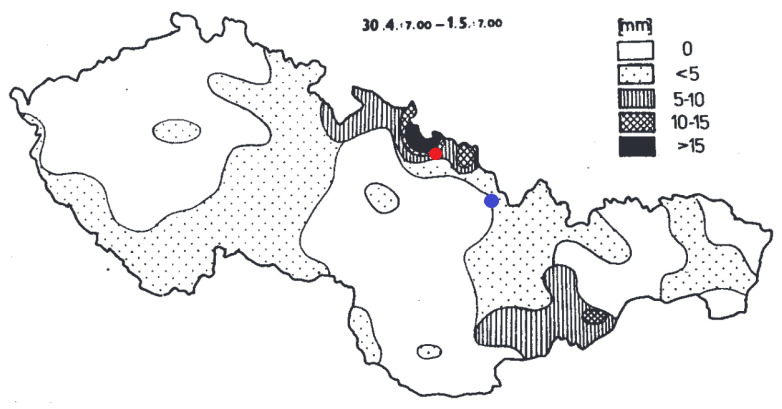
Precipitation in mm that fell on the area of the former Czechoslovakia in the time span from 30.4.1986 7AM CET to 1.5.1986 7AM CET shortly after the Chernobyl accident [6]. The red dot represents the hot spot in the Opava region, whereas the blue dot represents the second investigated area in the Ostravice river valley.

**Figure 2:**
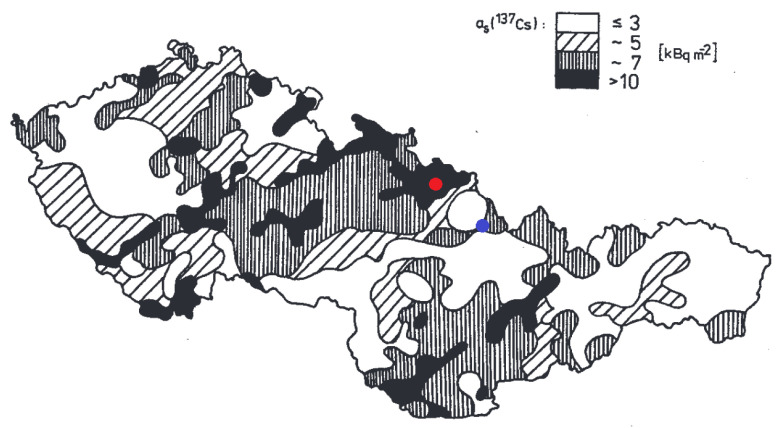
Distribution of the ${}^{137}$Cs surface activity on the area of the former Czechoslovakia after the Chernobyl accident in 1986 [6]. The red dot represents the hot spot in the Opava region whereas the blue dot represents the second investigated area in the Ostravice river valley.

In Figure 1 the precipitation in mm at the area of the former Czechoslovakia is depicted in the time span of 24 h on 30 April/1 May 1986, shortly after the Chernobyl accident. During that time, the radioactive cloud from the Chernobyl accident crossed the former Czechoslovakian border at the Opava region. It is clear that most of the precipitation fell on this area. In Figure 2 surface activities of ${}^{137}$Cs in the soil measured after the Chernobyl accident are presented.

As a consequence of high precipitation, the initial values of surface activities in the Opava region measured on 17 June 1986 [31], exceeded 10 kBq/m${}^{2}$ and spanned the interval from 23 kBq/m${}^{2}$ to 52 kBq/m${}^{2}$; whereas, in the Ostravice river valley, only 0.59 kBq/m${}^{2}$ were obtained, i.e., at least a 40-times lower value. It is interesting to point out that the Ostravice river valley (Staré Hamry) belongs to the network of localities where mushrooms are regularly checked for their ${}^{137}$Cs content by the National Radiation Protection Institute of the Czech Republic, while the hot spot area at the Opava region is not checked [32].

The collected mushrooms were cleaned, divided into caps and stipes and then sliced and dried for 4 days in air. After 4 days, they were dried in a laboratory dryer for 22 h at $105{\;}^{\circ }$C. The individual parts of the fruiting bodies were chopped in a blender and filled into 3 mL plastic vials that fit the well of a 30% relative efficiency low-background well-type HPGe spectrometer (GWD-3023, Baltic Scientific Instruments, Riga, Latvia).

The well detector dimensions were 16 mm in diameter and 50 mm in depth. The ultra-low background cryostat was made from ultra pure Al (5N5 AlSi 1%), OFE-OK electrolytic copper and its uranium and thorium content is less than 1 ppb. The detector was placed in a 10 cm lead shielding with an 8 mm radiopure copper liner. Activity of natural occurring radionuclides in the 2 cm inner chamber of the lead shielding was less than 5 Bq/kg.

The detector operates in a shallow underground laboratory at VŠB-Technical University of Ostrava, Czech Republic, at about 4 meters below the ground level. The resulting gamma background represents 0.0023 and 0.0029 cps in the regions of interest of 661.66 keV ${}^{137}$Cs and 1460.82 keV ${}^{40}$K gamma peaks, respectively.

**Figure 3:**
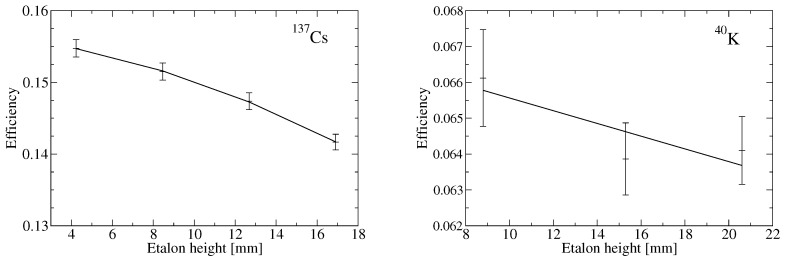
Efficiency calibration curves for different sample heights in the 3 mL vials for ${}^{137}$Cs (**left**) and for ${}^{40}$K (**right**) for the low-background well-type HPGe spectrometer GWD-3023.

As heights of the mushroom samples in the 3 mL vials differ and span the interval from 8 mm to 16 mm, the efficiency calibration for the ${}^{137}$Cs 661.66 keV gamma line was performed for four different heights of a standard ${}^{137}$Cs solution provided by Eurostandard, Czech Republic (4.3, 8.5, 12.7 and 17.0 mm). The efficiency curve for the GWD-3023 spectrometer as a function of the sample height was approximated by a quadratic function fitting the measured values obtained for the standard solutions thus enabled to determine the efficiency for an arbitrary sample height (see Figure 3). The resulting relative standard uncertainty of the efficiency introduced by the fitting procedure is less than 0.07%.

A similar procedure was applied to the efficiency calibration for the ${}^{40}$K 1460.82 keV gamma peak. Here, three samples of a powder 99.5% pure KCl provided by Penta, Czech Republic, of different heights were prepared (8.8, 15.3 and 20.6 mm), and the efficiency curve was approximated by a linear function (see Figure 3). The resulting relative standard uncertainty of the efficiency introduced by the fitting procedure is less than 1.2%.

In addition to ${}^{137}$Cs, there exists another radiocaesium isotope in nature, ${}^{134}$Cs. The activity of ${}^{134}$Cs can be calculated from the 604.72 keV peak. The specific activity ratio of ${}^{134}$Cs and ${}^{137}$Cs, $a_{134}/a_{137}$, can help to track the source of radiocaesium. Taking into account different half-lives of ${}^{137}$Cs and ${}^{134}$Cs, $T_{137}=30.08\left(9\right)$ year [33] and $T_{134}=2.0652\left(4\right)$ year [34], respectively, we can calculate the initial ratio of the specific activities of ${}^{134}$Cs and ${}^{137}$Cs, $a_{134_{0}}/a_{137_{0}}$, for April 1986 (the Chernobyl accident) under the assumption that all radiocaesium has the Chernobyl origin:

(1) $$a_{134_{0}}/a_{137_{0}}=a_{134}/a_{137}\times \exp\left[\ln2\times t\left(1/T_{134}-1/T_{137}\right)\right]\;,$$

where *t* is the time between the initial deposition and measurement. If the assumption is correct $a_{134_{0}}/a_{137_{0}}$ should coincide (within the error bars) with the reported Chernobyl experimental values 0.5–0.6 [35,36] and also with the value ${\left(a_{134_{0}}/a_{137_{0}}\right)}_{\exp}=0.515\left(15\right)$ calculated from the ratios of the surface activities of ${}^{134}$Cs and ${}^{137}$Cs in the Opava region measured on 17 June 1986 [31].

If the ratio obtained from Equation (1) is higher than the reported Chernobyl initial experimental value, this indicates an additional post-Chernobyl radiocaesium source; if it is lower, a pre-Chernobyl radiocaesium source plays a non-negligible role. This is the case of the second investigated area at the Ostravice river valley where the initial ratio of the surface activities of ${}^{134}$Cs and ${}^{137}$Cs measured on 17 June 1986 equals 0.22 [31]. Therefore, to analyse suspected additional non-Chernobyl sources of radiocaesium, it is useful to define a radiocaesium enhancement factor $F_{\mathrm{enh}}$:

(2) $$F_{\mathrm{enh}}=\frac{a_{134_{0}}/a_{137_{0}}}{{\left(a_{134_{0}}/a_{137_{0}}\right)}_{\exp}}$$

To determine the ratio $a_{134}/a_{137}$, a large amount of material is necessary in order to detect ${}^{134}$Cs after more than 20 years after the Chernobyl accident. The samples of *Xerocomus badius* containing both caps and stipes from the Chernobyl hot spot in the Opava area collected in October and November 2011 and in October 2012 underwent the same procedure as the small samples and finally were placed into a Marinelli beaker (volume 450 mL) and measured on the top of a 30% relative efficiency coaxial HPGe spectrometer (GC-3018, Canberra).

**Figure 4:**
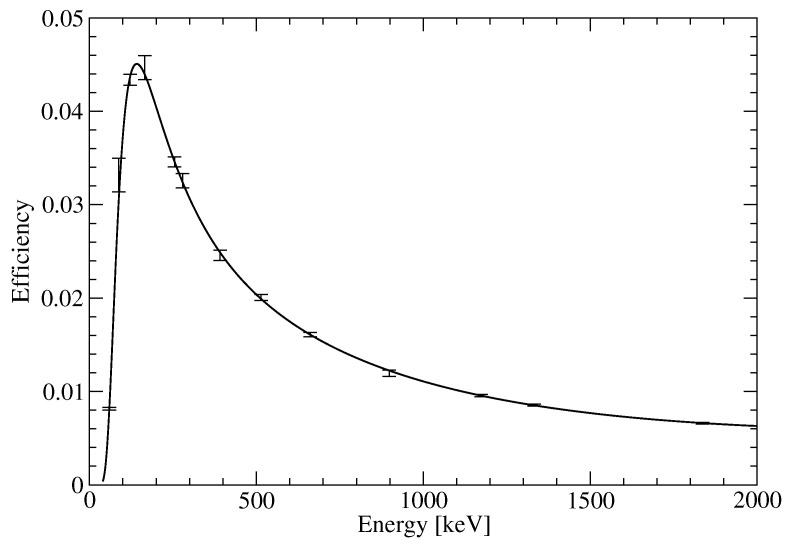
Efficiency calibration curve for the 30% relative efficiency coaxial HPGe spectrometer GC-3018 in the Marinelli geometry.

The detector was shielded by a massive shielding (100 mm Pb + 1 mm Cd + 1 mm Cu). The efficiency curve for the GC-3018 HPGe spectrometer in the Marinelli geometry was obtained from the MBSS2 standard containing isotopes ${}^{241}$Am, ${}^{109}$Cd, ${}^{57}$Co, ${}^{139}$Ce, ${}^{203}$Hg, ${}^{113}$Sn, ${}^{85}$Sr, ${}^{137}$Cs, ${}^{88}$Y and ${}^{60}$Co provided by Eurostandard, Czech Republic (see Figure 4).

The effect of selfabsorption was estimated for the GC-3018 HPGe spectrometer and the 3 mL vials and was found to represent less than 4% for ${}^{137}$Cs and less than 2% for ${}^{40}$K. Spectra of the samples were collected with and without calibration point sources provided by Eurostandard, Czech Republic, which were placed separately above each sample.

A ${}^{137}$Cs point source was used to determine the selfabsorption for the 661.66 keV gamma line and the gamma line of 1408.01 keV from a ${}^{152}$Eu point source was used to estimate the effect for the 1460.82 keV ${}^{40}$K gamma line. The selfabsorption effect for the well-type HPGe spectrometer decreases rapidly due to geometry of the well.

To estimate a committed effective dose *E* caused by the consumption of mushrooms containing a higher amount of ${}^{137}$Cs, the following formula can be applied [37]:

(3) $$E=m\times a_{137f}\times h_{137}\;,$$

where *m* is the annual intake of fresh mushrooms (kg per person), $a_{137f}$ the ${}^{137}$Cs specific activity of fresh mushrooms (Bq/kg), and $h_{137}$ stands for the conversion factor for ingestion intake of ${}^{137}$Cs ($1.3\times 10^{-8}$ Sv/Bq) [38].

## 3. Results and Discussion

Spectra of the samples in the 3 mL vials were measured in October 2016 using the low-background GWD-3023 HPGe spectrometer. Measurement times spanned the interval from 3.5 to 87 h. The obtained specific activities of ${}^{137}$Cs and ${}^{40}$K, recalculated for 1 November 2011 (middle of the collection period), are presented in Table 1. It is clearly seen that the specific activities of ${}^{137}$Cs are higher in the Opava region, while the specific activities of ${}^{40}$K are almost the same in both investigated areas.

A slightly higher level of both ${}^{137}$Cs and ${}^{40}$K is observed in the caps with an exception of *Armillariella mellea* in the Ostravice river valley for ${}^{137}$Cs. The highest values of the specific activity in Table 1 are close to the mean values for fruiting bodies of fungi in the Opole Anomaly collected in 2019 [39]. The Opole Anomaly is well known for extreme levels of ${}^{137}$Cs in Poland (surface activity exceeded 50 kBq/m${}^{2}$ in 1986) [40]. The Opole Anomaly is quite close to the Opava region investigated in this study.

Table 2 compares the ratios of the specific activities in caps and stipes in the two investigated localities. One can see that both *Russula ochroleuca* and *Xerocomus badius* are highly sensitive to the ${}^{137}$Cs soil content whereas caps of *Armillariella mellea* are about seven to eight times less sensitive, and its stipes are even 10- to 17-times less sensitive. Similar results were reported, e.g., in [16].

To estimate the strength of the linear relationship between the ratios of the specific activities of ${}^{137}$Cs and ${}^{40}$K (in Table 1 in the column $a_{137}/a_{40}$) for stipes and caps in both areas, a Pearson correlation test was applied. The Pearson correlation coefficient was equal to 0.93 and the *p*-value was less than 0.01 (p=0.0074) indicating a strong linear relationship between the ratios of the specific activities in agreement with [1], which supports the hypothesis that the transport of ${}^{137}$Cs from stipe to cap depends directly on ${}^{40}$K concentration for all three investigated species.

A mixed sample of the total dry weight of 29.591(15) g containing both caps and stipes of *Xerocomus badius* collected in October and November 2011 from the Chernobyl hot spot (Opava region) was measured in the Marinelli geometry in April 2012. The measurement time comprised 654,037 s. We found that the ${}^{137}$Cs specific activity $a_{137}=9400\left(200\right)$ Bq/kg is compatible with our results obtained from the measurement of the small samples of *Xerocomus badius* caps and stipes in the Opava region. The obtained ${}^{134}$Cs specific activity $a_{134}$ equalled 2.27(57) Bq/kg.

In October 2012, we collected caps of *Xerocomus badius* from the same place in the Opava region that underwent the same procedure as the previous sample and measured them for a longer time of 1,800,000 s. The initial ratios of the specific activities of ${}^{134}$Cs and ${}^{137}$Cs calculated from Equation (1) for both samples for April 1986 are summarized in Table 3 and compared to the initial reported Chernobyl experimental values in the Opava region.

The slightly higher values of the radiocaesium enhancement factor $F_{\mathrm{enh}}$ may indicate an additional contribution from the Fukushima accident in March 2011, but the final conclusion cannot be drawn because $F_{\mathrm{enh}}$ does not exceed 1 by more than 2σ.

The highest value of the ${}^{137}$Cs specific activity was observed for the species of *Xerocomus badius* (see Table 1) collected in the Opava region. Supposing the moisture content of mushrooms to be at 90% [17], the specific activity of the whole fresh mushroom is, in this case, at the value of 1119 Bq/kg, which already exceeds the limit in foodstuff recommended by IAEA (1000 Bq/kg fresh weight) [15].

The share of the ${}^{137}$Cs in the annual committed effective dose has been significantly increasing since the Chernobyl accident [41], and thus it is important to focus on mushroom consumers with special dietary habits.

The mean consumption of mushrooms calculated for the period 1986–2014 was 1.7 kg per year for the general population in the Czech Republic [41]. The annual consumption of wild mushrooms by dwellers has been estimated by Šišák [42] to be 7 kg per person. Based on Equation (3) the annual committed effective dose *E* for *Xerocomus badius* for dwellers equalled 0.102 mSv in the Opava region in 2011.

In the second examined location (the Ostravice river valley), the specific activity of ${}^{137}$Cs for the whole fresh mushroom $a_{137}=41.5$ Bq/kg resulted in the annual committed effective dose of E=0.004 mSv for dwellers. Therefore, the radiation risk in the Opava region is about 26 times higher. If we take into account a 50% decrease of the ${}^{137}$Cs activity due to cooking reported in [41], the annual committed effective dose becomes even lower.

## 4. Conclusions

The highest levels of ${}^{137}$Cs were found in caps of the species *Xerocomus badius* and *Russula ochroleuca* in the Opava region (11.8 kBq/kg and 8.77 kBq/kg, respectively). *Armillariella mellea* shows very low accumulation of radiocaesium in both locations. Furthermore, the dominant Chernobyl origin of radiocaesium at the hot spot in the Opava region was confirmed by means of the ${}^{134}$Cs/${}^{137}$Cs activity ratio. The linear relationship between the ratios of specific activities of ${}^{137}$Cs and ${}^{40}$K for stipes and caps was validated as well.

The potential risk from the consumption of *Xerocomus badius* in the Opava region is about 26-times higher than in the Ostravice river valley and represented the annual committed effective dose of 0.102 mSv at maximum in 2011. We also showed that the low-background well HPGe detector GWD-3023 equipped with ultra-low background shielding can be efficiently used for routine investigation of the ${}^{137}$Cs content in small mushroom samples with a dry weight of less than 1 g and a volume lower than 2–3 mL, which fit in the detector well. 

## Figures and Tables

**Table 1:** Specific activities of ${}^{137}$Cs and ${}^{40}$K, $a_{137}$ and $a_{40}$, for caps and stipes of the investigated dried mushrooms at the two localities and their ratio. Ratios of specific activities $a_{137}$ and $a_{40}$ in caps and stipes, $R_{137}$ and $R_{40}$, are also displayed. Calculated standard uncertainties are shown in parentheses.

Locality	Species	Part	$a_{137}$(Bq/kg)	$a_{40}$(Bq/kg)	$a_{137}/a_{40}$	$R_{137}$	$R_{40}$
Opava area	*Russula ochroleuca*	cap	8772(89)	1123(59)	7.81(42)	2.199(43)	1.15(11)
		stipe	3990(67)	975(76)	4.09(33)		
	*Xerocomus badius*	cap	11,810(160)	1250(160)	9.4(12)	1.132(22)	1.23(18)
		stipe	10,430(150)	1017(75)	10.26(77)		
	*Armillariella mellea*	cap	217.0(62)	1717(82)	0.1264(70)	2.129(98)	1.115(70)
		stipe	101.9(37)	1541(63)	0.0662(36)		
Ostravice area	*Russula ochroleuca*	cap	406.8(69)	1078(47)	0.377(18)	1.626(54)	1.119(95)
		stipe	250.2(7.2)	963(70)	0.260(21)		
	*Xerocomus badius*	cap	428.7(91)	1005(63)	0.427(28)	1.075(35)	1.27(14)
		stipe	398.8(97)	789(67)	0.505(45)		
	*Armillariella mellea*	cap	62.8(18)	1687(44)	0.0372(14)	0.962(40)	1.299(53)
		stipe	65.2(20)	1299(40)	0.0502(21)		

**Table 2:** Ratios of the specific activities $a_{137}$ and $a_{40}$ for caps and stipes in the investigated areas. Standard uncertainties are shown in parentheses.

Part	Species	$a_{137_{\mathrm{Opava}}}$/$a_{137_{\mathrm{Ostravice}}}$	$a_{40_{\mathrm{Opava}}}$/$a_{40_{\mathrm{Ostravice}}}$
Cap	*Russula ochroleuca*	21.56(43)	1.042(70)
	*Xerocomus badius*	27.54(69)	1.25 (18)
	*Armillariella mellea*	3.46(14)	1.018(56)
Stipe	*Russula ochroleuca*	15.95(53)	1.01(11)
	*Xerocomus badius*	26.15(74)	1.29(15)
	*Armillariella mellea*	1.563(73)	1.186(61)

**Table 3:** Initial ratios of $a_{134_{0}}/a_{137_{0}}$ calculated from Equation (1) for two samples of *Xerocomus badius* from the Opava region and the radiocaesium enhancement factors $F_{\mathrm{enh}}$ calculated from Equation (2) for the experimental initial ratio in the Opava region, ${\left(a_{134_{0}}/a_{137_{0}}\right)}_{\exp}=0.515\left(15\right)$. Standard uncertainties are shown in parentheses.

Sample Collection	$a_{134_{0}}/a_{137_{0}}$	$F_{\mathrm{enh}}$
October–November 2011	0.75(23)	1.46(45)
October 2012	0.74(17)	1.44(34)

## Data Availability

https://homel.vsb.cz/~ale02/mushrooms_life2021.xls, accessed on 4 December 2021.

## Abbreviations

The following abbreviations are used in this manuscript:

IAEA	International Atomic Energy Agency
GPS	Global Positioning System
CET	Central European Time
OFE-OK	Oxygen Free Copper of Very High Purity
HPGe	High Purity Germanium Spectrometer
